# Poster 2000: A case study in effective innovative strategic multi-modal recruitment strategies in difficult-to-recruit patient populations: study of safety and tolerability of novel SLIT in an adolescent population

**DOI:** 10.1186/1939-4551-7-S1-P17

**Published:** 2014-02-03

**Authors:** Anne Marie Salapatek, Justin Buck, Piyush Patel

**Affiliations:** 1 Inflamax Research, Mississauga, ON, Canada

## Background

Poor subject recruitment is a major cause of cost over-runs, extended finish dates, and delayed agency submissions. Failure to meet recruitment targets is particularly common in allergy immunotherapy studies where unique challenges such as allergen seasonality, lengthy study durations and studying allergen specific populations are necessary. Historically, standard modalities of advertising in newspaper, radio, and television have been employed with mixed success. Increasing availability of communication and sharing technologies, and social media availabilities, affords innovative approaches and tools to customize the recruitment of subjects, especially difficult populations.

## Methods

A dose escalation study investigated the safety and tolerability of a novel sub-lingual immunotherapy in subjects aged 12-17yrs old for the treatment of house dust-mite allergy (HDM). Several challenges to the recruitment of adolescents were overcome including: tight study inclusion criteria, summer vacations, conflicting parent & adolescent schedules, confounding seasonal allergens, and the beginning of a new school year within two months from the study start-up. After thorough identification of HDM allergy symptoms, an appropriate advertising message was created and approved by the sponsor and IRB for use in all advertising campaigns and modalities. A cutting-edge model has been developed with a Business-To-Consumer (B2C) marketing mindset. A similar model to our proprietary Online Central Recruitment Update Platform was used where ideal subject profiles are constructed and advertising avenues are specifically chosen to ensure high penetration into the target demographic.

## Results

A mix of traditional plus B2C advertising strategies led to successful recruitment mid-September 2013 and on schedule database soft-lock. 160 subjects were booked for screening, 129 screened, 37 screen-passed and dosed, and 36 completed the study with 35 per protocol completes. The mean age of the subjects passing all inclusion/exclusion criteria and completing the study was 15.3±0.5; male to female ratio was 3:2.

## Conclusions

Strategic planning of marketing and advertising campaigns using a blend of carefully selected traditional and contemporary modalities, targeted to a well-defined niche demographic, yielded successful recruitment of an HDM allergic adolescent population.

